# Typing FGFR2 translocation determines the response to targeted therapy of intrahepatic cholangiocarcinomas

**DOI:** 10.1038/s41419-021-03548-4

**Published:** 2021-03-11

**Authors:** Xiaohong Pu, Qing Ye, Jing Cai, Xin Yang, Yao Fu, Xiangshan Fan, Hongyan Wu, Jun Chen, Yudong Qiu, Shen Yue

**Affiliations:** 1 Department of Pathology, Nanjing Drum Tower Hospital, The Affiliated Hospital of Nanjing University Medical School, Nanjing, 210008 Jiangsu Province China; 2 Department of Pathology, Division of Life Sciences and Medicine, The First Affiliated Hospital of USTC, University of Science and Technology of China, Hefei, Anhui 230036 China; 3 Division of Life Sciences and Medicine, Intelligent Pathology Institute, University of Science and Technology of China, Hefei, Anhui 230036 China; 4 Department of Medical Genetics, Nanjing Medical University, Nanjing, 211166 Jiangsu Province China; 5 Department of Hepatopancreatobiliary Surgery, Nanjing Drum Tower Hospital, The Affiliated Hospital of Nanjing University Medical School, Nanjing, 210008 Jiangsu Province China

**Keywords:** Targeted therapies, Bile duct cancer

## Abstract

Chromosomal translocations involving *fibroblast growth factor receptor 2* (*FGFR2*) gene at the breakpoints are common genetic lesions in intrahepatic cholangiocarcinoma (ICC) and the resultant fusion protein products have emerged as promising druggable targets. However, predicting the sensitivity of *FGFR2* fusions to FGFR kinase inhibitors is crucial to the prognosis of the ICC-targeted therapy. Here, we report identification of nine *FGFR2* translocations out of 173 (5.2%) ICC tumors. Although clinicopathologically these *FGFR2* translocation bearing ICC tumors are indistinguishable from the rest of the cohort, they are invariably of the mass-forming type originated from the small bile duct. We show that the protein products of *FGFR2* fusions can be classified into three subtypes based on the breaking positions of the fusion partners: the classical fusions that retain the tyrosine kinase (TK) and the Immunoglobulin (Ig)-like domains (*n* = 6); the sub-classical fusions that retain only the TK domain without the Ig-like domain (*n* = 1); and the non-classical fusions that lack both the TK and Ig-like domains (*n* = 2). We demonstrate that cholangiocarcinoma cells engineered to express the classical and sub-classical fusions show sensitivity to *FGFR*-specific kinase inhibitors as evident by the suppression of MAPK/ERK and AKT/PI3K activities following the inhibitor treatment. Furthermore, the kinase-deficient mutant of the sub-classical fusion also lost its sensitivity to the FGFR-specific inhibitors. Taken together, our study suggests that it is essential to determine the breakpoint and type of *FGFR2* fusions in the small bile duct subtype of ICC for the targeted treatment.

## Background

Cholangiocarcinoma is a highly heterogeneous epithelial tumor arising from the biliary tract^1^. Data from the United States have shown that the incidence of cholangiocarcinoma has steadily increased over the past three decades and its 5-year survival rate is below 10%^2^. The estimated 5-year survival rate could be even lower in China. Surgical resection followed by transplantation is the only option for patients with early-stage tumors^3^. Due to the insidious onset of the illness, most patients have reached the advanced stage of the disease when clinical symptoms present^4^. Systemic non-targeted therapies that are extrapolated from those commonly used in other gastrointestinal malignancies show limited effects in progressive cholangiocarcinomas^5^. According to the fifth World Health Organization Digestive System Tumors Classification^6^, cholangiocarcinomas can be classified into intrahepatic cholangiocarcinoma (ICC), perihilar cholangiocarcinoma (PHCC), and distal cholangiocarcinoma (DCC) based on the anatomical location of the tumor within the biliary tree. Based on the gross examination, ICC can be further divided into four categories: mass-forming (MF) type, periductal-infiltrating (PI) type, intraductal growth (IG) type, and mixed pattern. Small duct ICC is mainly located in the peripheral parts of the liver and primarily shows a MF pattern, whereas large duct ICC is primarily located in juxtaposition to the liver hilum and spreads along the large portal tracts with a PI pattern. In addition to having different anatomical origins, these subtypes also have distinct epidemiology, risk factors, pathogenesis, and treatment^7,8^.

Recently, many groups have shown that chromosomal translocations with breakpoints at *fibroblast growth factor receptor 2* (*FGFR2*) frequently occur in ICC but not PHCC and DCC^9,10^. The full-length FGFR2 protein consists of an extracellular region, three immunoglobulin (Ig)-like domains, a single hydrophobic transmembrane segment, and two cytoplasmic tyrosine kinase (TK) domains^11^. Numerous studies have demonstrated that *FGFR2* fusions promote tumorigenesis due to their inappropriately activated kinase activity^10^. Activated FGFR signals primarily through mitogen-activated protein kinase (MAPK) and phosphoinositide-3-kinase (PI3K)/AKT/mTOR with assistance from an adapter protein FGFR substrate 2 (FRS2), which also activates signal transducers such as phosphorylation of PDK, and also regulated by multiple downstream substrates^12–14^. Different partners of *FGFR2* gene fusions have also been demonstrated in recent studies of ICC patients. Bicaudal C Homolog 1 (BICC1) has been identified as the most frequent fusion partner in this entity, with rare cases demonstrating *FGFR2*-*AHCYL1*, *FGFR2*-*MGEA5*, *FGFR2*-*TACC3*, *FGFR2*-*KIAA1598*^8–10,15–23^. These fusions not only determine the anatomic location of the tumors, but also are proved as the driven factors of ICC^10^. Results from phase II trials of Infigratinib (BGJ398), a FGFR2 kinase inhibitor, exhibited manageable toxicity and significant clinical effect on chemotherapy-refractory cholangiocarcinoma containing *FGFR2* fusions^24^. Also, another FGFR inhibitor Pemigatinib was granted accelerated approval by the Food and Drug Administration for cholangiocarcinoma with an FGFR2 fusion or rearrangement in the United States^25^. Despite these impressive progresses, the specific mechanism of different fusions to respond to the FGFR-targeted drugs in tumor cells remains to be determined.

In contrast to primary sclerosing cholangitis in North America and hepatitis C infection in Japan, the possible risk factor for ICC in China is hepatitis B viral infection^26–29^. Geographical and ethnic variations in the ICC epidemiology suggest an involvement of both genetic and environmental factors. In a previous study, we found that the incidence of *FGFR2* translocation in a cohort of Chinese ICC patients was much lower than that in other countries^30^, and no prognostic utility and gender trends were observed. When we enlarged the number of patients, we found that the incidence of *FGFR2* translocation was even lower, and the fusion partners and breakpoints were different from reported studies. Since different fusions may indicate different prognoses and treatments, these translocation-specific immunoprofiles and biological behaviors should be distinguished.

In the present study, we identified *FGFR2* translocation in a group of 173 patients with ICC. Screened by RNA sequencing and confirmed by Sanger sequencing, we explored several unreported *FGFR2* gene breakpoints and fusion patterns. Through in vitro study, we found that as long as fusion chimeras contain the *FGFR2* kinase domain, they respond to the targeted drugs. MAPK and AKT that are downstream of FGFR signaling are activated in ICC cell lines harboring chimeras containing *FGFR2* kinase domain. Selective FGFR-targeted drugs inhibited the phosphorylation of ERK and AKT, suggesting that both MAPK and AKT pathways participate in tumorigenesis.

## Materials and methods

### Clinical samples and clinicopathological data

Clinical specimens of ICC were provided by Nanjing Drum Tower Hospital. In this study, 173 tumors diagnosed with ICCs in radical resections were retrieved from the diagnostic files and consultation cases in the Department of Pathology between 2005 and 2017. Medical records of preoperative information, including the general information, HBV infection, cholangiolithiasis, hepatic steatosis, and schistosome infection, were tabulated for all patients (Table 1). The pathological data, such as tumor number, maximum dimension, gross classification, differentiation, and histological type, were recorded. Tumor TNM staging was determined according to the eighth edition of the American Joint Committee on Cancer/Union for International Cancer Control TNM Classification and Stage Groups for ICC. Because of the scattered large geographic location of the patients and long follow-up duration, only 80.3% (139/173) of patients in the research contained follow-up information.

**Table 1:** Clinicopathologic characteristics of FGFR2 translocation and non-translocation in ICCs.

No. of patients	*FGFR2* translocation (*N* = 9)	*FGFR2* non-translocation (*N* = 164)	*p* value
Average age (year)	57.00 (48–62)	61.27 (34–81)	0.054
Male/female	6/3	79/85	0.324
HBV infection (+/–)	5/4	45/119	0.123
Cholangitis (+/–)	7/2	123/41	1.000
Fatty liver (+/–)	2/7	29/135	0.664
Schistosome (+/–)	0/9	7/157	1.000
Tumor numbers (*n*)	3.25 (1–10)	2.11 (1–11)	0.467
Tumor maximum dimension (cm)	7.04 (2.8–11.5)	5.66 (0.5–16)	0.266
Gross classification			0.038^a^
MF type	9	110	
Non-MF type	0	54	
Histological classification			0.052
Pancreaticobiliary type	8	92	
Others type	1	72	
Differentiation (W/M/P/U)	0/5/4/0	4/75/82/3	0.893
G (0/1/2/3/4)	2/4/1/1/1	16/79/52/15/2	0.119
S (0/1/2/3/4)	4/2/2/1/0	31/63/45/8/17	0.287
T (I/II/III/IV)	5/4/0/0	56/64/35/9	0.310
Stage (I/II/III/IV)	5/3/0/1	51/46/26/41	0.288
OS (month)	28.25 (4–53)	45.10 (3–82)	0.984
DFS (month)	30.48 (4–53)	33.37 (1–82)	0.263

*G* grade, *M* moderately differentiated, *OS* overall survival, *P* poorly differentiated, *S* stage, *T* tumor, *U* undifferentiated, *W* well differentiated.

^a^Statistically significant.

### Fluorescent in situ hybridization

To identify *FGFR2* rearrangements, break-apart fluorescent in situ hybridization (FISH) was performed on formalin-fixed paraffin-embedded (FFPE) tumors using BAC clones corresponding to the 5’ (RP11-34I13, CTD-2529K22, CTD-2014E7) and 3’ (RP11- 879C17, RP11-454I6, CTD-2160A22) sequences flanking the *FGFR2* gene and labeled by nick translation in green and red, respectively. FISH was performed on 2-μm thick sections.

Before hybridization, slides were deparaffinized, dehydrated in 100% ethanol, and air-dried. Sections were digested in 5 mg/ml, pH 2.0 pepsin for 5–50 min and fixed in 1% formaldehyde and phosphate-buffered saline at room temperature, then dehydrated in 70, 80, and 100% ethanol. Probes were described above. Denaturation (5 min at 85 °C) and hybridization (overnight at 37 °C) were carried out in the Hybridizer (DAKO, Denmark). The procedure was followed by a post-wash using 0.4 × SSC and 2 × SSC. Diamidinophenylindole was used as a counterstain. Slides were scored for hybridization signals using Olympus BX51 (Olympus, Japan) with a filter set including diamidinophenylindole single bandpass (counterstain), orange single bandpass, and green bandpass. In order to be considered positive, separate Spectrum Orange and/or Spectrum Green signals had to be present in greater than 20% of nuclei throughout the tumor^10^.

### Immunohistochemistry

Representative 4-μm serial sections of the tumor were prepared from 10% FFPE tissue blocks for immunohistochemistry. Briefly, all slides were exposed to 3% hydrogen peroxide for 10 min to block endogenous peroxidase activity. FGFR2 antibody (#AB58201, 1:300, anti-mouse, Abcam, USA), ERK (#4695, 1:250, anti-rabbit, Cell signaling technology, USA), P-ERK (#4370, 1:250, anti-rabbit, Cell signaling technology, USA), AKT (#4685, 1:200, anti-rabbit, Cell signaling technology, USA), P-AKT (#4060, 1:100, anti-rabbit, Cell signaling technology, USA) incubated with tumor sections in a humidified chamber at 4 °C overnight, followed by the secondary anti-mouse peroxidase-conjugated secondary antibody (EnVision^TM^ Detection Kit, DAKO, Denmark) or anti-rabbit peroxidase-conjugated secondary antibody (EnVision^TM^ Detection Kit, DAKO, Denmark) at 37 °C for 30 min.

The IHC score was calculated by multiplying the staining intensity (0 = no staining, 1 = mild staining, 2 = moderate staining, and 3 = strong staining) by the percentage of immunoreactive tumor cells (0–100). The immunostaining result was considered to be 0 or negative when the score was <25; 1+ or weak when the score was 26–100; 2+ or moderate when the score was 101–200; or 3+ or strong when the score was 201–300.

### Analysis of the whole transcriptome sequence data

Nine translocation cases proved by FISH were analyzed by RNA sequencing. Total RNA from FFPE samples was extracted after xylene deparaffinization using the RNeasy formalin-fixed paraffin-embedded kit (QIAGEN, Dalian, China). Complementary DNA (cDNA) libraries composed of 150–200 bp inserts were prepared from 2 μg of total RNA using the TruSeq RNA Sample Preparation Kit (Illumina, San Diego, CA). The libraries were subjected to paired-end sequencing of 50–100 bp fragments on the HiSeq2000 instrument (Illumina Inc., San Diego, CA, http://www.illumina.com). Fusion Catcher (version 0.99.4e) was used with parameters (BLAT aligner, otherwise, the default parameter was used) that apply the Bowtie aligner to perform both transcriptome and genome mapping and then used the BLAT aligner to further map unmapped reads and count fusion supporting evidence.

### Sanger sequencing of the FGFR2 fusion transcripts

PCR of *FGFR2* fusion transcripts: total RNA was reverse-transcribed into cDNA using SuperScript III (Life Technologies, Carlsbad, CA). The cDNA was subjected to PCR amplification using Ex Taq (Takara Bio, Tokyo, Japan) and specific primers (Supplementary Table 1).

The PCR products were directly sequenced by Sanger sequencing using the BigDye terminator kit (Life Technologies) and an ABI Basecaller (Applied Biosystems, Grand Island, NY).

### cDNA cloning

cDNAs of full-length *FGFR2* fusion chimeras were isolated from the corresponding tumor specimens by RT-PCR using PrimeSTAR GXL polymerase (Takara Bio) and specific primers (Supplementary Table 2). Each cDNA was subcloned into a pRK5 vector containing an N-terminal Flag tag (Cell Biolabs, San Diego, CA) using homologous recombination kit (ClonExpress^®^ Entry, Nanjing, China) to generate a plasmid expressing the fusion protein with a FLAG epitope tag.

### Site-directed mutagenesis

The kinase-dead mutant was constructed by replacing tyrosine with phenylalanine at amino acid 564 of FGFR2 in the fusion gene BF494 (abbreviation to *BICC1(exon 1-3)-FGFR2(exon10-18)* fusion) using a site-directed mutagenesis kit (Takara Bio).

### Recombinant lentivirus construction

Each cDNA was subcloned into a GLV2-CMV-EGFP-MCS-PGK-Puro vector (Cell Biolabs, San Diego, CA) to generate recombinant lentivirus expressing the fusion protein with a FLAG epitope tag.

### Cell culture

Human cholangiocarcinoma cell lines HCCC-9810 or RBE were cultured in RPMI 1640 (WISENT INC.) medium supplemented with 10% heat-inactivated fetal bovine serum (FBS) (Gibco), 1% penicillin/streptomycin (Gibco). Cells were grown as monolayer cultures and maintained in a humidified atmosphere with 5% CO_2_ at 37 °C. Mouse NIH3T3 fibroblast cells were maintained in DMEM with 10% FBS. Ba/F3, murine interleukin-3 dependent pro-B cells were maintained in RPMI 1640 with 10% FBS, 1% penicillin/streptomycin, and 5 ng/ml mouse IL3 in 5% CO_2_ at 37 °C.

### Proliferation assays

Exponentially growing Ba/F3 cells were electroporated by the Nucleofector TM 2b Device (Lonza) with pRK5 or pRK5-FGFR2 fusion chimeras expressing plasmids. After 24 h, cells expressing different chimeras were distributed into 96-well plates with or without IL3 as indicated, placing 10^4^ cells in 100 μL media into each well. Cell growth was evaluated by CCK8 assay (#K1018, APExBIO, USA) on days 0, 1, 2, 3, and 7. After incubation with CCK8 (1:10) for 2 h, cells were counted by reading the absorbance at 450 nm using a Microplate reader (SpectraMax iD5, Molecular Devices, USA). Each sample had at least three duplicate wells and was independently performed in triplicate.

### Colony formation of FGFR2 fusions

Mouse NIH3T3 fibroblast cells were transfected with indicated FGFR2 fusion chimeras using Fugene HD (Promega). A total of 200 cells were plated in a 6-well. On day 12, the wells were fixed with 4% paraformaldehyde (PFA), stained with crystal violet and counted.

### Transwell migration assay

RBE and HCCC-9810 grown in 6-well plates were transfected with FGFR2 fusion chimeras or their mutants. Transwell migration assay was performed using transwell inserts (MCEP24H48, Millipore) with a filter of 8 μm pore. A total of 2.5 × 10^4^ cells in serum-free medium were seeded into the upper chamber of the insert and complete medium was added to the lower chamber. After 24 h incubation, the cells were fixed with 4% PFA and stained with crystal violet. Then cells on the top surface of the membrane were wiped off, and cells on the lower surface were examined with microscope at 100× magnification. Four random fields were photographed for counting and the average number of migrated cells was used as a measure of migration capacity.

### Cell viability assay

RBE and HCCC-9810 were infected with recombinant lentiviruses expressing FGFR2 fusion chimeras or their mutants, then were distributed into 96-well plates with indicated concentrations of BGJ398 (#T1975, TargetMol, USA) or AZD4547 (#T1948, TargetMol, USA). After 72 h treatment, cell viability was evaluated by CCK8 assay as described above.

### Small-interfering RNAs-mediated FGFR2 depletion

FGFR2-specific small-interfering RNA (siRNA) was chemically synthesized (sense: AGCCCUGUUUGAUAGAGUAUATT, and antisense: UAUACUCUAUCAAACAGGGCUTT, Sangon Biotech). Cells were seeded at 1 × 10 ^6^ cells in 6-well plates and 5 µl of siRNA (20 pmol/μl) was transfected using Lipofectamine RNAiMAX (Thermo Fisher Scientific).

### Immunoblot analysis

Transfected RBE cells were serum-starved for 2 h, after which vehicle (DMSO) or indicated compounds were added for a further 2 h. The cells were lysed in RIPA buffer for western blotting. The primary antibodies were antibodies against FLAG tag (#F1804, Sigma, USA), Phospho-FRS2-α (Tyr196) (#3864, Cell Signaling Technology), ERK (#4695, Cell Signaling Technology), phospho-p44/42 ERK(Thr202/Tyr204) (#9106, Cell Signaling Technology), AKT1 (#2967, Cell Signaling Technology), and phospho-AKT (Ser473) (#4051, Cell Signaling Technology).

### Statistical analysis

All data analyses were performed using SPSS 19.0 software (SPSS Inc., Chicago, IL). Fisher’s exact test was used for categorical data, and the Student *t*-test was used for continuous data. Analysis of variance or the Kruskal–Wallis rank sum test was used to compare differences among different groups. The χ^2^ or Fisher’s exact test was utilized for comparison of ratios. Patient post-resection survival was estimated by the Kaplan–Meier method with a log rank test. Differences were considered to be statistically significant when *p* values were less than 0.05.

## Results

### Clinicopathological characteristics of patients carrying FGFR2 translocations

**Fig. 1:**
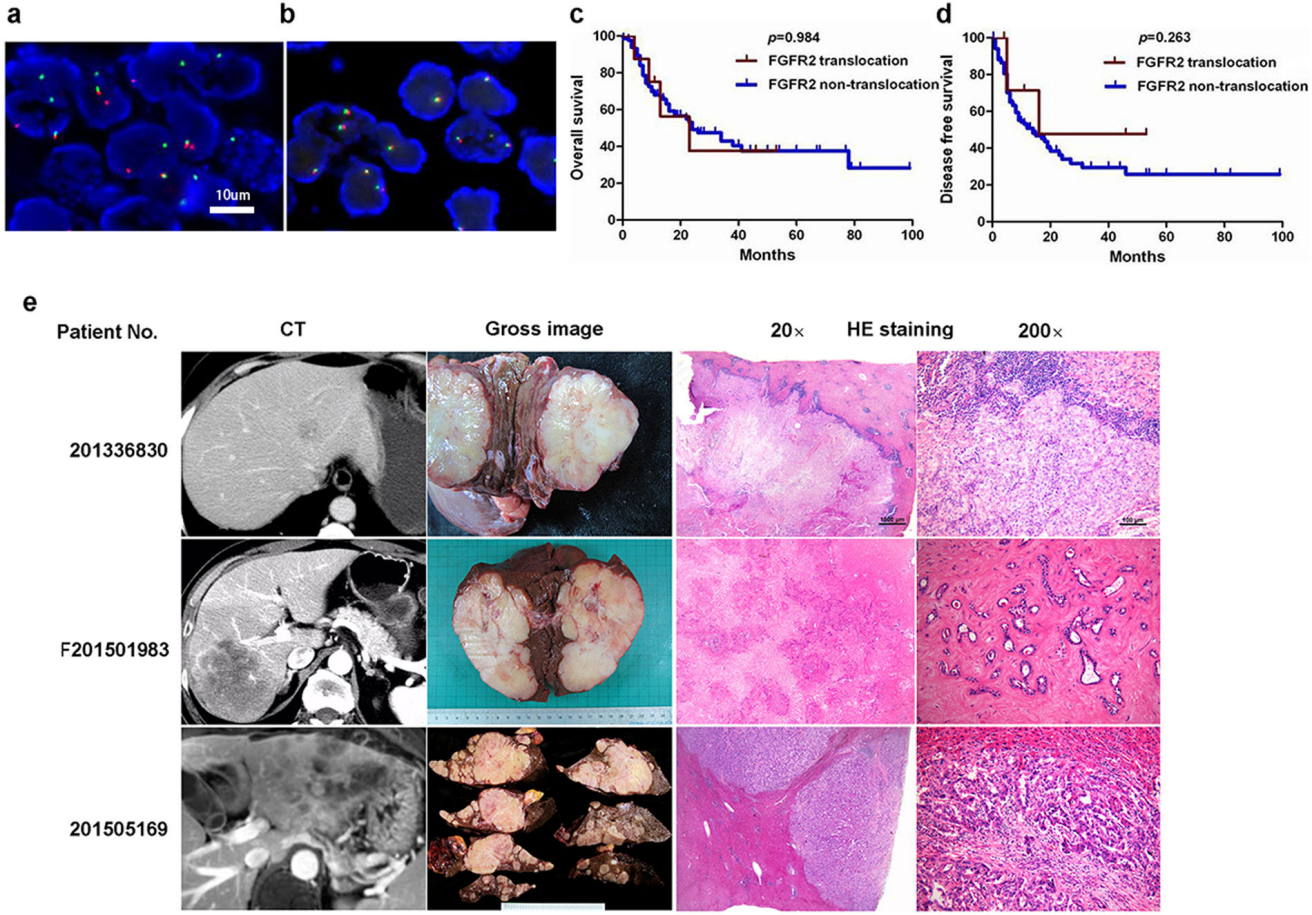
Clinical subtypes in cholangiocarcinoma. Green and red spots indicate the genomic location of 5’ and 3’ FISH probes for the *FGFR2* gene. Distinct orange and green signals in more than 20% of the tumor cells represent *FGFR2* rearrangement. Schematic representation of *FGFR2* gene translocation (**a**) and non-translocation (**b**). Overall survival curve (**c**) and disease-free survival curve (**d**) stratified by *FGFR2* translocation and non-translocation cases in ICCs (Kaplan–Meier method). The radiologic, gross and histopathologic (20× and 200×) images of three representative patients whose tumor possessed an *FGFR2* translocation (**e**). Three ICCs, which respectively numbered No. 201336830, No. F201501983, and No. 201505169 showed small, large, and multiple tumors, in radiologic or gross morphology, were all mass-forming (MF) type. For histological classification, these were all pancreaticobiliary type adenocarcinoma as presented in 20× and 200× HE staining.

A total of 173 ICC patients were included in this study. Through FISH testing, *FGFR2* translocations were identified in nine (5.2%) tumor specimens (Fig. 1a, b). There was no significant difference in age, risk factors, tumor number and size, tumor differentiation, clinical stage, and prognosis between translocation-positive and -negative cases (Table 1). Same as in the North American study^9^, we found that *FGFR2* translocations presented in younger patients (*p* = 0.054), and the ICC harboring the translocations were histologically classified as pancreaticobiliary type (*p* = 0.052). However, different from other studies^27^, due to the low incidences of *FGFR2* translocation and limited follow-up information, *FGFR2* translocation carriers did not show differences in overall survival and disease-free survival (Fig. 1c, d). In our study, all of the tumors with *FGFR2* translocations showed MF pattern (*p* = 0.038). Radiological, gross anatomical, and histopathologic (magnification 20× and 200×) images of three representative patients whose tumors possessed an *FGFR2* translocation are shown in Fig. 1e.

### Identification of FGFR2 breakpoints and fusion partners

**Fig. 2:**
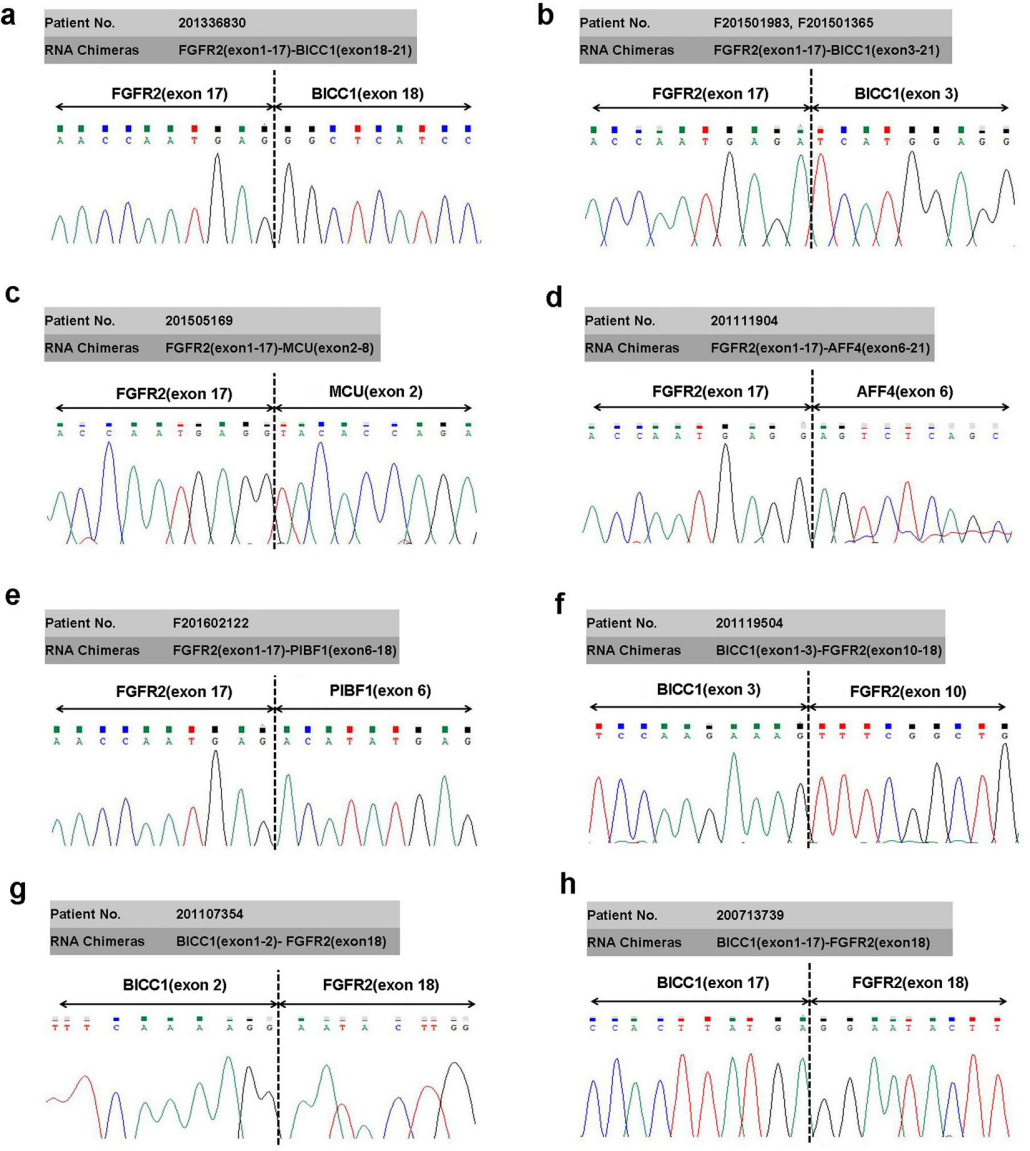
Sanger sequencing of the RT-PCR product validates in-frame fusion transcripts. Fusion transcripts were detected as *FGFR2(exon 1-17)-BICC1(exon 18-21)* (**a**), *FGFR2(exon 1-17)-BICC1(exon 3-21)* (**b**), *FGFR2(exon 1-17)-MCU(exon 2-8)* (**c**), *FGFR2(exon 1-17)-AFF4(exon 6-21)* (**d**), *FGFR2(exon 1-17)-PIBF1(exon 6-18)* (**e**), *BICC1(exon 1-3)-FGFR2(exon10-18)* (**f**), *BICC1(exon 1-2)-FGFR2(exon18)* (**g**), and *BICC1(exon 1-17)-FGFR2(exon18)* (**h**).

Whole transcriptome high-throughput sequencing of tumor specimens is one of the most effective methods for screening fusion oncogenes. To find novel molecular alterations, nine specimens carrying *FGFR2* translocations were sequenced by massively parallel paired-end transcriptome sequencing, and eight fusions chimeras were identified. The sequence reads spanning the junctions of eight fusion candidates were amplified by RT-PCR using primers as indicated (Supplementary Table 2) and the breakpoint sequence was determined by Sanger sequencing. This analysis identified *FGFR2(exon 1-17)-BICC1(exon 18-21)*, *FGFR2(exon 1-17)-BICC1(exon 3-21)*, *FGFR2(exon 1-17)-MCU(exon 2-8), FGFR2(exon 1-17)-AFF4(exon 6-21)*, *FGFR2(exon 1-17)-PIBF1(exon 6-18)*, *BICC1(exon 1-3)-FGFR2(exon10-18)*, *BICC1(exon 1-2)-FGFR2(exon18)* and *BICC1(exon1-17)-FGFR2(exon18)* (Fig. 2). Except for *FGFR2(exon 1-17)-BICC1(exon 3-21)*, the other seven fusion chimeras have not been reported previously. The information of all of the *FGFR2* fusion partners (novel in our studies and reported in other researches) in cholangiocarcinoma is listed in Supplementary Table 3.

### Analysis of the fusion chimeras

According to the breakpoints and fusion modes, we classified these eight fusion chimeras as classical, sub-classical, and non-classical fusions.

**Fig. 3:**
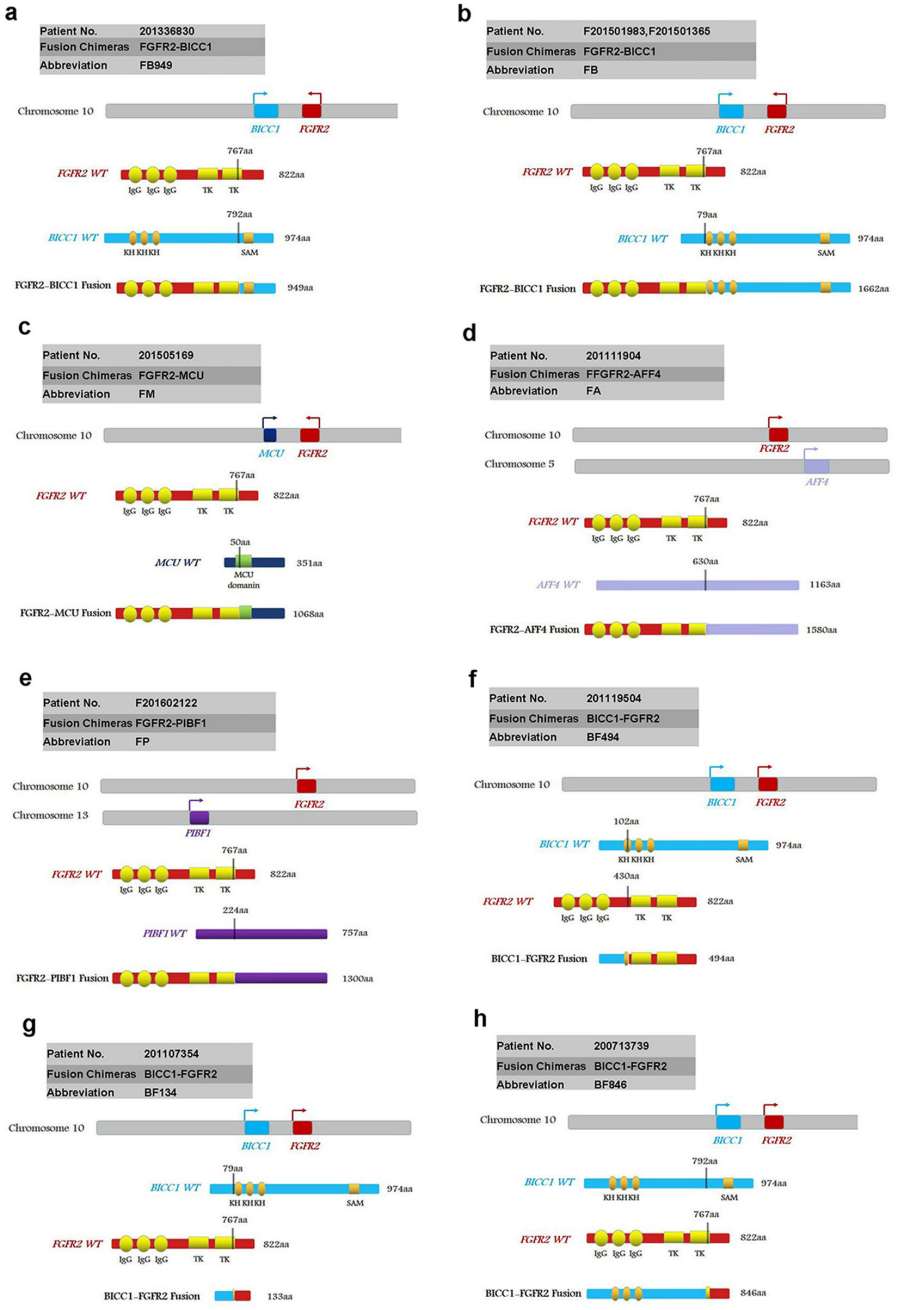
Schematic representation of *FGFR2* fusion proteins. Fusion proteins were described as FB949 (**a**), FB (**b**), FM (**c**), FA (**d**), FP (**e**), BF494 (**f**), BF134 (**g**), and BF846 (**h**). Ig immunoglobulin-like domain, TM transmembrane domain, kinase protein tyrosine kinase domain, CC coiled-coil domain, KH K homology RNA binding domain, SAM sterile alpha motif. The vertical lines indicate breakpoints.

Classical: classical fusion chimeras have Ig-like domains and TK domains of *FGFR2*, sharing the same fusion mode consisting *FGFR2* amino terminus (1-767aa) and the carboxyl terminus of the partners. The classical fusion chimeras we found were described as FB949 (abbreviation to *FGFR2(1-767aa)-BICC1(792-974aa)*) (Fig. 3a), FB (abbreviation to *FGFR2(1-767aa)-BICC1(79-974aa)*) (Fig. 3b), FM (abbreviation to *FGFR2(1-767aa)–MCU(50-351aa)*) (Fig. 3c), FA (abbreviation to *FGFR2(1-767aa)-AFF4(630-1163aa)*) (Fig. 3d), and FP (abbreviation to *FGFR2(1-767aa)-PIBF1(224-757aa)*) (Fig. 3e).

Sub-classical: fusion chimera BF494 (abbreviation to *BICC1 (exon 1-3)-FGFR2(exon10-18)*) (Fig. 3f) was assigned as sub-classical chimera, which contains the TK domains but not Ig-like domains of *FGFR2*.

Non-classical: the chimeras completely missing the Ig and TK domains and only maintaining the C-terminal tail of *FGFR2* were assigned as non-classical chimeras. BF134 (abbreviation to *BICC1(1-79aa)-FGFR2 (767-822aa)*) (Fig. 3g) and BF846 (abbreviation to *BICC1(1-79aa)-FGFR2 (767-822aa)*) (Fig. 3h) were classified as non-classical fusions.

It is curious whether these different types of fusions exhibit different effect on ICC tumorigenesis and sensitivity on targeted drugs. *FGFR2*, *BICC1*, and *MUC* are all located on the long arm of chromosome 10^11,31,32^, while *AFF4* and *PIBF1* are located on the long arm of chromosome 5 and chromosome 13, respectively^33,34^. Based on the previous researches^10^, we speculate that these classical chimeras are drivers of ICC and sensitive to the *FGFR2*-targeted drugs. Since these non-classical or sub-classical fusion chimeras totally or partly miss those function domains, we are curious of their function in ICC formation.

### FGFR2 fusion chimeras promote cell proliferation and transformation

**Fig. 4:**
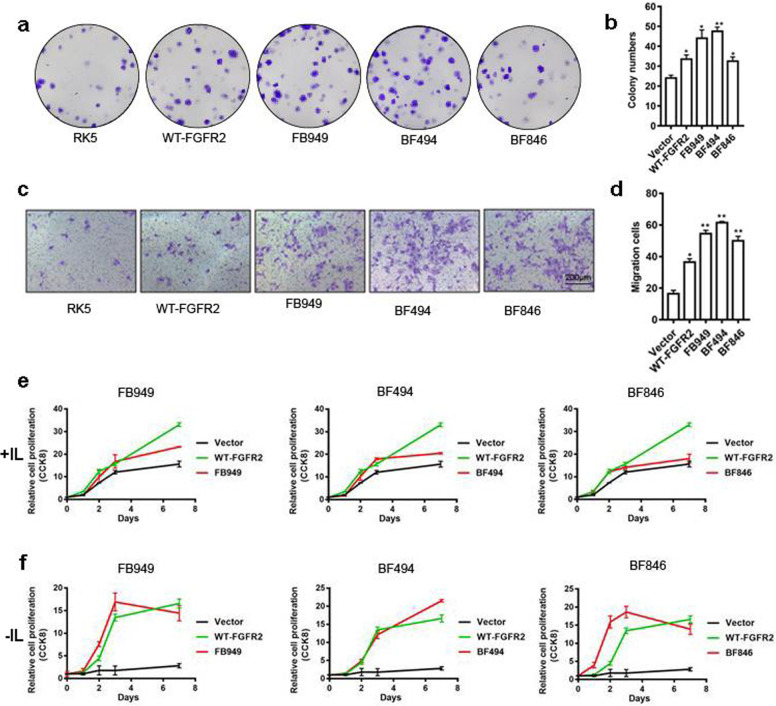
Proliferation and transformation activity of FGFR2 fusions. Representative images of colonies expressing different FGFR2 fusions in NIH3T3 cells are shown (**a**). Average colonies with different FGFR2 fusions are plotted (**b**). Representative images of transwell migration expressing different FGFR2 fusions in RBE cells are shown (scale bar = 100 μm) (**c**). Average numbers of migrated cells with FGFR2 fusions are plotted (**d**). Ba/F3 cell growth curve with (**e**) or without IL3 (**f**) are shown. *p < 0.05, **p < 0.01.

To assess the function of novel fusions identified in our ICC specimens and determine their responsiveness to *FGFR2*-targeted drugs, we constructed expression vectors of these fusions and labeled them with FLAG epitope tag (Supplementary Fig. 1). These fusion chimeras were then introduced into NIH3T3 cells for determining their transforming activity by colony formation assays (Fig. 4a). All fusions produced higher foci formation than the vector control (Fig. 4b and Supplementary Fig. 2). We also introduced these fusions into ICC cell lines RBE and HCCC-9810 for assaying their ability to induce cell migration. Transwell migration assay revealed that all three types of fusions promoted migration of ICC cells (Fig. 4c, d and Supplementary Fig. 3). Finally, to determine the oncogenic potential of these fusions, we introduced them into Ba/F3 cells, a line of murine cells that are dependent on IL3 for viability and growth. The results indicated that the fusions were able to sustain IL3-independent growth of Ba/F3 cell (Fig. 4e, f and Supplementary Figs. 4 and 5). Taken together, these data indicate that the FGFR2 fusions we identified have the propensity to promote tumorigenesis and metastasis of ICC.

### FGFR2 fusion chimeras show different sensitivities to FGFR-selective small-molecule kinase inhibitors (SMKIs)

**Fig. 5:**
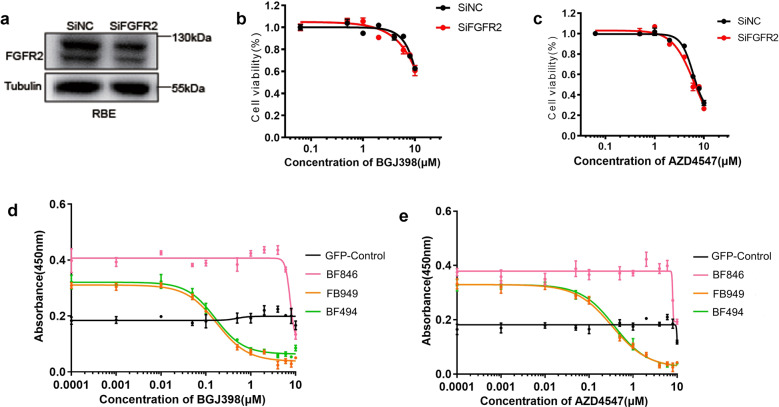
Response of RBE cells expressing *FGFR2* fusions to *FGFR2*-selective SMKIs BGJ398 and AZD4547. siRNA was used to interfere the expression of endogenous FGFR2 (**a**). siRNA interference did not change the response of RBE cells to BGJ398 (**b**) or AZD4547 (**c**). Dose-response curves for BGJ398 (**d**) and AZD4547 (**e**) in RBE cells. The viability of RBE cells expressing different FGFR2 fusions was detected by CCK8 assay after treatment with indicated concentration of BGJ398 or AZD4547.

To determine if the fusions are capable of rendering ICC cells sensitive to targeted therapy, we took advantage of HCCC-9810 and RBE cells, which have intact FGFR2 genes, and are known to be resistant to FGFR-specific TK inhibitors BGJ398 and AZD4547. To alleviate the interference of endogenous FGFR2 in evaluating the sensitivity to FGFR inhibitors, we silenced the expression of endogenous FGFR2 using siRNA. This manipulation of endogenous FGFR2 did not change the viability of RBE cells in response to either BGJ398 or AZD4547 treatment (Fig. 5b, c), suggesting that these cells are not dependent on endogenous FGFR2 for survival. We then infected RBE with lentiviruses expressing FGFR2 fusions corresponding to FB949, BF494, and BF846 representing classical, sub-classical, and non-classical *FGFR2* fusion, respectively. FACS analysis of GFP positive cells showed that more than 70% of RBE cells were infected successfully. Cells expressing FB949 and BF494 were highly sensitive to BGJ398 (Fig. 5d) and AZD4547 (Fig. 5e) compared with GFP control. It is reported that IC50 of BGJ398 is 1.4 nM in cell-free assay^35^, and IC50 of AZD4547 is 2.5 nM in cell-free assay^36^. The RBE cells expressing FB949 were highly sensitive (IC50 70.8 nM to BGJ398 and IC50 113.3 nM to AZD4547), ones expressing BF494 were also sensitive (IC50 69.5 nM to BGJ398 and IC50 130.2 nM to AZD4547), whereas ones expressing BF846 were resistant (IC50 > 1 μM) (Fig. 5d, e and Supplementary Table 4). BGJ389 and AZD4547 treatment in HCCC-9810 cells expressing representative FGFR2 fusions showed the similar results (Supplementary Fig. 6 and Supplementary Table 4). These results suggested that FGFR-selective SMKIs could only suppress the growth of ICC cells carrying the fusions containing TK domain.

### FGFR-selective SMKIs suppress the phosphorylation of AKT and ERK

**Fig. 6:**
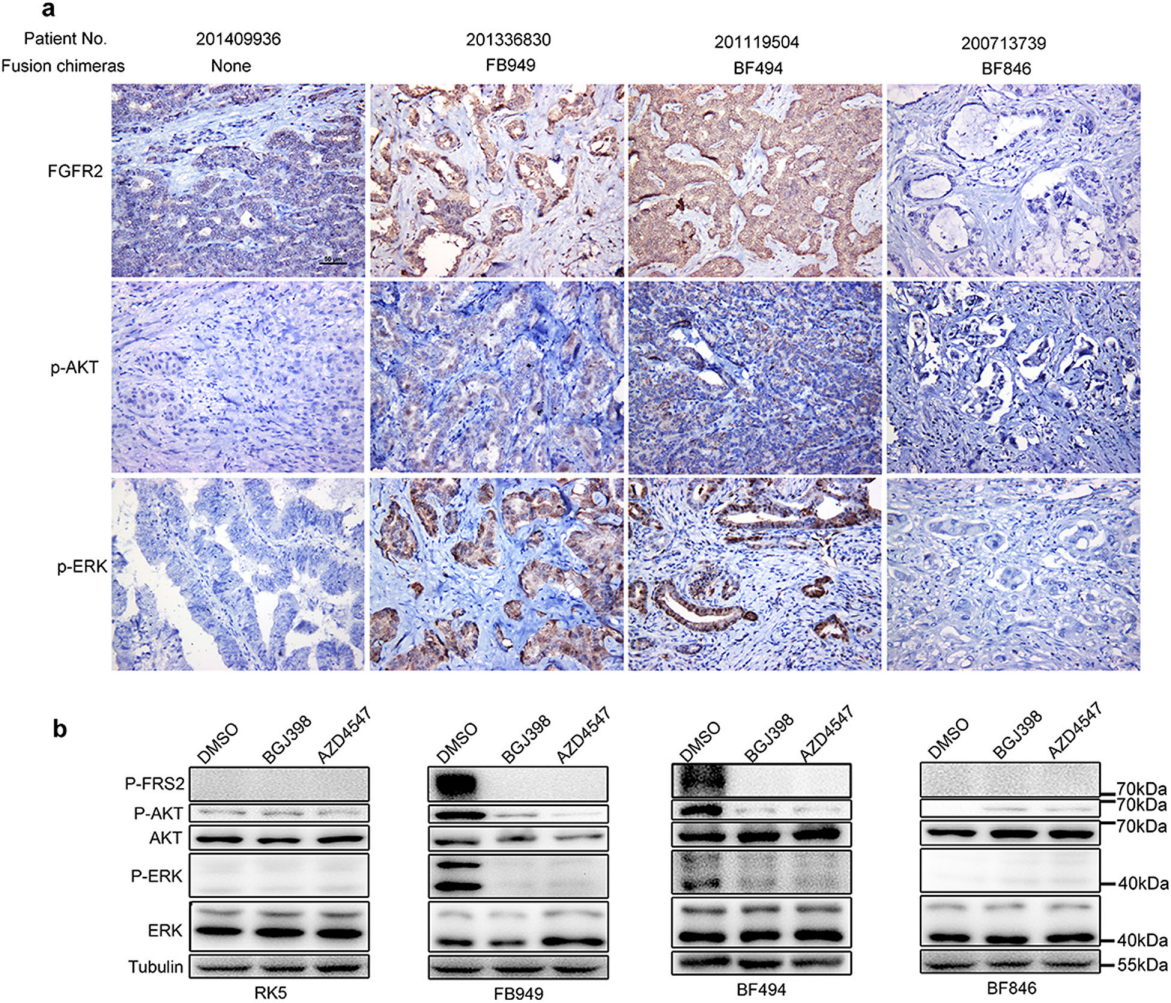
*FGFR2*-selective SMKIs suppressing the activation of *FGFR2* downstream pathway in RBE cells harboring sub-classical or classical chimeras. Representative images of FGFR2, p-AKT, and p-ERK immunostaining in tumor specimens harboring different FGFR2 fusion chimeras are shown (**a**). Tumors harboring classical (FB949) or sub-classical (BF494) fusions showed high-expression of FGFR2 and high phosphorylation of AKT and ERK. Western blotting showing signaling inhibition upon BGJ398 and AZD4547 treatment (**b**). Phosphorylation of FRS2, AKT, and ERK was detected in RBE cells expressing FB949, BF494, BF846, or vector control.

To investigate the mechanism of FGFR-targeted therapy, downstream FGFR signaling components were analyzed in ICC tissues and cell lines. Pre-treatment evaluation by immunohistochemistry demonstrated increased expression of p-ERK, p-AKT in tumors carrying classical, and sub-classical fusion chimeras, without increase of FGFR2, ERK, and AKT expression (Fig. 6a). However, due to the small sample size of *FGFR2* translocation, those differences were not significant between the *FGFR2* non-fusion and fusion groups (Table 1). Western blotting revealed that ICC cells expressing classical or sub-classical chimeras showed the phosphorylation of FRS2, ERK, and MAPK compared with control cells (Fig. 6b), which was suppressed by BGJ398 and AZD4547. These results indicated that the FGFR2 fusion proteins carrying *FGFR2* kinase domain could activate multiple downstream pathways, including MAPK/ERK and PI3K/AKT pathways. FGFR-selective SMKIs target the *FGFR2* fusion chimeras through suppressing the activation of downstream pathways.

### TK domain determines the sensitivity of FGFR2 fusion chimeras to selective SMKIs

**Fig. 7:**
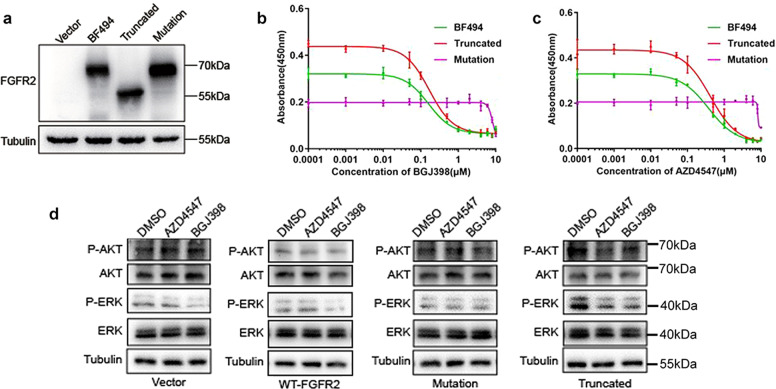
Response of RBE cells expressing BF494 variations to *FGFR2*-selective SMKIs BGJ398 and AZD4547. The expression of BF494, BF494-truncated, and BF494-mutation chimeras was detected by western blot with anti-Flag antibody (**a**). Dose-response curves for BGJ398 (**b**) and AZD4547 (**c**) in RBE cells expressing different BF494 variations. Western blotting showing phosphorylation of AKT and ERK in RBE cells expressing WT-FGFR2, BF494 truncation, BF494 mutation, or vector control (**d**).

Since the non-classical fusions missing the TK domain of FGFR2 and do not response to FGFR-selective SMKIs, we speculate that TK domain is essential for selective SMKIs responses. To further verify the function of TK domain in SMKIs sensitivity, we constructed two mutants based on the sub-classical fusion BF494. One is truncated at the 770th amino acid of FGFR2, containing the kinase domain but not C-terminal domain of BF494, named as BF494-truncated (truncated for short). The other mutant, named as BF494-P564 (mutation for short), was mutated at V564 site in TK domain (Fig. 7a), because the FGFR2 fusion chimeras harboring such mutation were reported to be resistant to FGFR inhibition^37^. Our results noted that chimeras missing the C-terminus of BF494 still show sensitivity to selective SMKIs, whereas chimeras mutated in the TK domain could not respond to FGFR2 inhibitors compared with the BF494 in RBE cells (Fig. 7b, c). Besides, our data also showed that the cells expressing V564F mutant of BF494 had reduced cell proliferation, compared to ones expressing wild type and truncated BF494. We speculate that the gatekeeper mutation changes the conformation of BF494, thereby inducing a spatial conflict with BGJ398 in its FGFR2-binding pocket and reducing kinase activity as well. They showed the similar results in HCCC-9810 cells as well (Supplementary Fig. 7). Truncated BF494 showed the phosphorylation of ERK, and MAPK that was suppressed by BGJ398 and AZD4547, whereas kinase-dead mutation could not activate MAPK/ERK and PI3K/AKT pathways (Fig. 7d). These results suggested that the TK domain of FGFR2 fusion chimeras is essential for the response to selective SMKIs.

## Discussion

*FGFR2* translocations, which are present in 5–38% of ICCs, represent driver mutations and predict tumor sensitivity to specific FGFR inhibitors in cholangiocarcinoma^8–10,15–19,21^. However, different geographical and ethnic variations in the ICC epidemiology suggest that *FGFR2* translocation may have different incidence rates and variations in different areas^38^. Our studies based on a cohort of Chinese patients showed that *FGFR2* fusions have many partners and breakpoints that were not reported previously and *FGFR2* translocation was only found in 9 of 173 patients with 5.2% incidence rate. Different from other studies, there was no significant difference between *FGFR2* translocation and non-translocation ICCs in clinicopathological features including gender, risk factors, stage, survival, and so on, mostly because of the low incident of *FGFR2* translocation. In North American study, *FGFR2* translocation was associated with enhanced survival, younger cohort, and female preponderant^9^, whereas in Japanese cohort, no survival or gender differences were noted but associated with hepatitis B and C virus infection^10^. Nevertheless, in our study, we found that tumors carrying *FGFR2* translocation were all of MF type (*p* = 0.038) gross classification. According to its macroscopic appearance, the Liver Cancer Study Group of Japan has subdivided ICC into four categories: MF type, PI type, IG type, and mixed pattern^7,8^. ICC deriving from malignant transformation of ductules and small bile ducts usually ends preferentially in a MF type, whereas ICC arising within second order of intrahepatic bile ducts or segmental bile ducts usually results in PI or IG type^6^. Clinical features and biological behaviors of ICC differ among the different macroscopic types and *FGFR2* translocation seems one of these differences in our study. Thus, it is beneficial for selecting *FGFR2* translocation patients before molecular target treatment based on its macroscopic appearance. For immunoprofile, still no significant difference with the non-translocation group due to limited cases. Therefore, IHC is not a useful way to distinguish the fusion cases to all ICCs, and only next-generation sequencing could precisely tell us the fusion mode of *FGFR2* that is very important for the follow-up target treatments.

In the present study, four fusion partners, two *FGFR2* breakpoints, and a total of three fusion modes were found. Except for *BICC1*, other three fusion partners *MCU, AFF4*, and *PIBF1* were all first reported. Based on breakpoints and fusion modes, we divided these chimeras into three subtypes. The classical fusion is remaining all of the functional domains, sub-classical fusion maintaining the TK domain, but missing the Ig-like domains and non-classical fusion only containing the C-terminal tail of *FGFR2*. Classical fusions have been reported in a series of ICC cohorts, followed with frequent of *FGFR2*-*BICC1* and rare of *FGFR2*-*TACC3*, *FGFR2*-*MGEA5*, *FGFR2*-*KIAA1598*, *FGFR2*-*AFF3*, and so on^10,16–22^. Although the transcripts of AHCYL1-FGFR2 and BICC1-FGFR2 missing the kinase domain of FGFR2 were reported in Japanese population, these fusions were reciprocal products of chromosomal translocations generating the *FGFR2-BICC1* and *FGFR2-AHCYL2* oncogenic drivers and cannot be regarded as the non-classical fusions^10^. In North American population, one case harboring FGFR2-FRK fusion was reported^23^, the fusion is also listed as non-classical in our research due to its loss of the FGFR2 kinase domain. Sub-classical fusions were not reported before.

**Fig. 8:**
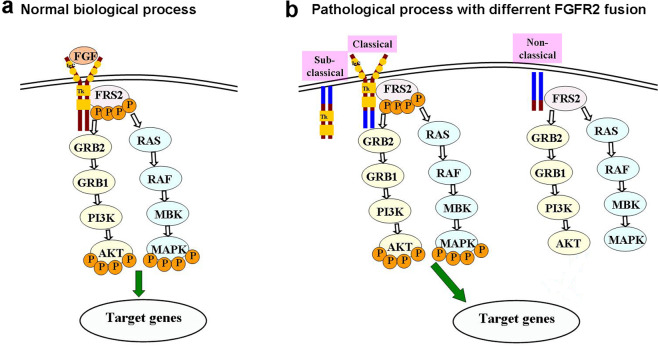
Models for the signaling transduction of fusion chimeras and targeted drugs working in the downstream of FGF signal pathway. Normal biological processes of FGF signal pathway (**a**). Sub-classical and classical chimeras lead to formation of a fusion protein consisting of a transcription factor fused to an FGFR kinase domain with consequent FGFR dimerization and activation leading to ligand-independent FGF signaling. Non-classical fusion cannot active downstream signal pathway. SMKIs suppressed the phosphorylation of FRS2, AKT, and MAPK then confer anchorage-independent growth and in vivo tumorigenesis (**b**).  represents Ig-like domain of *FGFR2*,  represents tyrosine kinase domain of *FGFR2*,  represents phosphorylation, and  represents stimulating target gene activity.

In normal biological processes, The FGF-FGFR axis is activated by binding of FGF to FGFR, which activates the FGFR TK with resultant autophosphorylation of tyrosine in the special residues. Phospho-FGFR then phosphorylates adapter proteins, including FRS2^11^. FGFR preferentially signals through FRS2 to the MAPK and AKT pathway, ultimately regulates multiple downstream substrates^19^ (Fig. 8a). Although FGFR2 fusion partners in sub-classical and classical chimeras have a wide range of original functions, the fusions engage the homodimerization and therefore force catalytic activation of adjacent FGFR2 kinase domains. In our study, both the phosphorylation of AKT and MAPK were activated in cell lines harboring classical and sub-classical fusions. Non-classical chimeras missing the kinase domain fail to activate the downstream signal pathways (Fig. 8b).

BGJ398 and AZD4547 are both orally bioavailable, selective, ATP-competitive selective FGFR kinase inhibitors showing vigorous suppressing activity against tumor models harboring FGFR alterations^16,35,36^. On the Phase II study of BGJ398 in patients with FGFR-altered advanced cholangiocarcinoma, BGJ398 showed 18.8% overall response rate and 83.3% disease control rate in patients carrying *FGFR2* fusion with manageable toxicities actively supporting its further biologic and clinical investigation^24^. However, in this clinical trial *FGFR2* translocation did not precisely detect the breakpoints and fusion modes. Consistent with other studies, our results showed that BGJ398 and AZD4547 had strong efficiency in *FGFR2* classical and sub-classical fusions but not in non-classical fusions. Therefore, not all of the ICC patents carrying *FGFR2* fusion apply to target treatment, the fusion mode is critical to determine the effectiveness. In sub-classical and classical fusion, FGFR-selective SMKIs totally block the phosphorylate activation of all the downstream signaling, therefore block signal transduction and activation. Non-classical fusions missing the function site for phosphorylating activation showed no effects to FGFR-selective SMKIs. Therefore, we expected that FGFR-selective SMKIs are suitable for the treatment of ICCs carrying *FGFR2* classical or sub-classical fusions.

In this study, we found that ICCs with *FGFR2* translocation occurred only in MF type, which may indicate different mechanism in variant gross classifications and helpful for select target patients before molecular treatments. Another striking result from the current comprehensive analysis of the *FGFR2* translocation was that there were three different *FGFR2* fusion modes in ICCs based on the Chinese population. We identified that not all of the fusion chimeras response to targeted therapy and the efficacy of FGFR-selective SMKIs was determined by the breakpoints and fusion modes not fusion partners of *FGFR2*. Only classical and sub-classical fusions retaining TK domain can respond to *FGFR2*-selective SMKIs by repressing the phosphorylation of ERK and AKT. So, it is essential to detect the breakpoints and fusion modes of *FGFR2* in certain gross classifications before targeted treatment.

## Supplementary information

supporting information

supplemental figure 1

supplemental figure 2

supplemental figure 3

supplemental figure 4

supplemental figure 5

supplemental figure 6

supplemental figure 7
