# Supplementary material for: Typing FGFR2 translocation determines the response to targeted therapy of intrahepatic cholangiocarcinomas
Source: Cell Death Dis. 2021 Mar 11;12(3):256. doi: 10.1038/s41419-021-03548-4 (PMC7946919; doi:10.1038/s41419-021-03548-4)
Supplement: Supplementary file 1 — supporting information [file 41419_2021_3548_MOESM1_ESM.docx]

Supporting information

**Typing *FGFR2* translocation determines the response to targeted therapy of intrahepatic cholangiocarcinomas**

Xiaohong Pu, Qing Ye, Jing Cai, Xin Yang, Yao Fu, Xiangshan Fan, HongYan Wu, Jun Chen, Yu-Dong Qiu and Shen Yue

Supplemental Table 1: Primers for Sanger sequencing of the fusion transcripts;

Supplemental Table 2: Primers for cloning the fusion chimeras;

Supplemental Table 3: Information of FGFR2 fusion partners in Cholangiocarcinoma;

Supplemental Table 4: IC50 values of different fusion chimeras to target drugs;

Figure legends of S1-S7.

**Supplementary Table 1 Primers for Sanger sequencing of the fusion transcript**

| **Patients No.** | **Fusion chimera** | **Primer-F(5’-3’)** | **Primer-R(5’-3’)** | **Product length** |
| --- | --- | --- | --- | --- |
| 200713739 | BICC1(EXON1-17)-  FGFR2(EXON18 ) | CCACAAATACCTGGAGTG | TCAGGGTAACTAGGTGAAT | 164bp |
| 201107354 | BICC1(EXON1-2)-  FGFR2(EXON18) | AGGGAAAGGCAGAAGTGG | TCAGGGTAACTAGGTGAAT | 89bp |
| 201119504 | BICC1(EXON1-3)-  FGFR2(EXON10-18) | TGGAGGAAACAAATACGC | AGTTCATACTCGGAGACCC | 200bp |
| 201111904 | FGFR2(EXON1-17)-  AFF4(EXON6-21) | ATGATGAGGGACTGTTGG | TGCCCATTTGAAGTTTTA | 169bp |
| F201501365 F201501983 | FGFR2(EXON 1-17) -BICCI(EXON 3-21) | GTGATGTCTGGTCCTTCG | ATCTGCGTATTTGTTTCC | 277bp |
| F201602122 | FGFR2(EXON1-17)-  PIBF1(EXON 6-18) | CGTTTGGCCTTAGAATTA | TAATTCTAAGGCCAAACG | 100bp |
| 201336830 | FGFR2(EXON1-17)-  BICCI(EXON 18-21) | GAAGCCCTGTTTGATAGA | ATAGTGTTCCGTTGATTTG | 451bp |
| 201505169 | FGFR2(EXON1-17)-  MCU(EXON2-8 ) | TGACACTGGGTACGTGGTT | TTCTGCCAGGAAGCGATC | 287bp |

**Supplementary Table 2 Primers for cloning the fusion chimeras**

| **Patients No.** | **Fusion chimeras** | **Primer-F(5’-3’)** | **Primer-R(5’-3’)** | **Ligase** | **Fusion chimeras length** |
| --- | --- | --- | --- | --- | --- |
| 200713739 | BICC1(EXON1-17)-  FGFR2(EXON18 ) | CAGAGGAGGATCTGATCGATGATGGCCGCCCAGGGAGAGCC | CTTCTGCAGGTCGACTCTAGATCATGTTTTAACACTGCCGTT | ClaI xbalI | 2541bp |
| 201107354 | BICC1(EXON1-2)-  FGFR2(EXON18) | CAGAGGAGGATCTGATCGATGATGGCCGCCCAGGGAGAGCC | CTTCTGCAGGTCGACTCTAGATCATGTTTTAACACTGCCGTT | ClaI xbalI | 402bp |
| 201119504 | BICC1(EXON1-3)-  FGFR2(EXON10-18) | CAGAGGAGGATCTGATCGATGATGGCCGCCCAGGGAGAGCC | CTTCTGCAGGTCGACTCTAGATCATGTTTTAACACTGCCGTT | ClaI xbalI | 1485 bp |
| 201111904 | FGFR2(EXON1-17)-  AFF4(EXON6-21) | CAGAGGAGGATCTGATCGATGATGGTCAGCTGGGGTCGTTT | TCAAGATATCAACTTGGCATTCTAGATCATGTTTTAACACTGCCGTT | ClaI xbalI | 4743bp |
| F201501365 F201501983 | FGFR2(EXON 1-17) -BICCI(EXON 3-21) | ATTCCCCGGGGATCCTCTAGAATGGTCAGCTGGGGTCGTTT | CTACCAGCGGCCACTGACACTCTAGACTTCTGCAGGTCGAC | xbalI | 4989 bp |
| F201602122 | FGFR2(EXON1-17)-  PIBF1(EXON 6-18) | CGATGAATTCCCCGGGGATCCATGGTCAGCTGGGGTCGTTT | CTAGGTCTTCATCTTTTGTTAAGCTTCTGCAGGTCGGACTC | BamHI HindIII | 3903 bp |
| 201336830 | FGFR2(EXON1-17)-  BICCI(EXON 18-21) | CGATGAATTCCCCGGGGATCCATGGTCAGCTGGGGTCGTTT | ATGGCGGCCAAGCTTCTGCAGCTACCAGCGGCCATGACA | BamHI PstI | 2850 bp |
| 201505169 | FGFR2(EXON1-17)-MCU(EXON2-8 ) | CAGAGGAGGATCTGATCGATGATGGTCAGCTGGGGTCGTTT | TCAATCTTTTTCACCAATTTGTCTAGATCATGTTTTAACACTGCCGTT | ClaI xbalI | 3207bp |

**Supplemental Table 3 Information of FGFR2 fusion partners in Cholangiocarcinoma (novel in our studies and reported in other research)**

| **Partner** | **Full name** | **Gene locus** | **Introduction** | **References** | **Cohort** | **Number** |
| --- | --- | --- | --- | --- | --- | --- |
| *MCU* | 窗体顶端  [Mitochondrial calcium uniporter](https://www.ncbi.nlm.nih.gov/gene/90550) | 10q22.1 | Encoding a calcium transporter that localizes at the mitochondrial inner membrane, which is necessary to mitochondrial calcium uptake | Novel | ICC | 1/173 |
| *AFF4* | AF4/FMR2 family member 4 | 5q.31.1 | Encoding protein belongs to the AF4 family of transcription factors involved in leukemia. | Novel | ICC | 1/173 |
| *PIBF1* | 窗体顶端 [Progesterone immunomodulatory binding factor 1](https://www.ncbi.nlm.nih.gov/gene/10464) 窗体底端 | 13q21.33-13q22.1 | Encoding a progesterone-induced-blocking factor, which is induced by the steroid hormone progesterone. PIBF1 regulates multiple aspects of the immune system to promote normal pregnancy including cytokine synthesis, natural killer (NK) cell activity, and arachidonic acid metabolism. PIBF1 was also reported to promote the proliferation, migration and invasion of glioma. | Novel | ICC | 1/173 |
| *BICC1* | Bicaudal C Homolog 1 | 10q21.1 | Encoding an RNA-binding protein that is active in regulating gene expression by modulating protein translation during embryonic development. A sterile alpha motif (SAM) at the carboxyl terminus of BICC1 is an interaction and dimerization module. | 10  11  12  18  13 | ICC  Cholangiocarcinoma  ICC  ICC  Cholangiocarcinoma | 2/66  1/6  1/28  41/107  2/not mention |
| *TACC3* | Transforming acidic coiled-coil containing protein 3 | 窗体顶端  4p16窗体顶端  4p16.3  窗体底端  窗体底端 | 窗体顶端  Encoding a member of the transforming acidic coiled-coil protein family. The encoded protein is a motor spindle protein that may play a role in stabilization of the mitotic spindle. This protein may also play a role in growth and differentiation of certain cancer cells. | 11  12 | Cholangiocarcinoma  ICC | 1/6  1/28 |
| *AHCYL1* | 窗体顶端 Adenosylhomocysteinase like 1 窗体底端 | 窗体顶端  1p13.3  窗体底端 | 窗体顶端  Encoding protein interacts with inositol 1,4,5-trisphosphate receptor, type 1 and may be involved in the conversion of S-adenosyl-L-homocysteine to L-homocysteine and adenosine | 10 | ICC | 7/66 |
| *MGEA5* | 窗体顶端  Meningioma expressed antigen 5  窗体底端 | 窗体顶端  10q24.32  窗体底端 | 窗体顶端  Encoding protein catalyzes the dynamic modification of cytoplasmic and nuclear proteins by O-linked N-acetylglucosamine (O-GlcNAc) addition and removal on serine and threonine residues | 11 | Cholangiocarcinoma | 1/6 |
| *KIAA1598* | Shootin 1 | 窗体顶端  10q25.3  窗体底端 | Also known as shootin 1 | 12 | ICC | 1/28 |
| *PPHLN1* | 窗体顶端 Periphilin 1 窗体底端 | 窗体顶端  12q12  窗体底端 | 窗体顶端  Encoding protein that sequentially incorporated into the cornified cell envelope during the terminal differentiation of keratinocyte at the outer layers of epidermis. This protein interacts with periplakin, which is known as a precursor of the cornified cell envelope. The cellular localization pattern and insolubility of this protein suggest that it may play a role in epithelial differentiation and contribute to epidermal integrity and barrier formation. Multiple alternatively spliced transcript variants encoding distinct isoforms have been observed. | 18 | ICC | 17/107 |
| *CREB5* | 窗体顶端  cAMP responsive element binding protein 5  窗体底端 | 窗体顶端  7p15.1  窗体底端 | 窗体顶端  Encoding protein specifically binds to CRE as a homodimer or a heterodimer with c-Jun or CRE-BP1, and functions as a CRE-dependent trans-activator. Alternatively spliced transcript variants encoding different isoforms have been identified. | 16 | Cholangiocarcinoma | Not mention |
| *CCDC6* | 窗体顶端  coiled-coil domain containing 6  窗体底端 | 窗体顶端  10q21.2  窗体底端 | 窗体顶端  Encoding protein is ubiquitously expressed and may function as a tumor suppressor. A chromosomal rearrangement resulting in the expression of a fusion gene containing a portion of this gene and the intracellular kinase-encoding domain of the ret proto-oncogene is the cause of thyroid papillary carcinoma  窗体底端 | 17 | ICC | 2/55 |
| *WAC* | 窗体顶端  WW domain containing adaptor with coiled-coil  窗体底端 | 窗体顶端  10p12.1  窗体底端 | 窗体顶端  Encoding protein contains a WW domain which found in a wide range of signaling proteins. This domain mediates protein-protein interactions and binds proteins containing short linear peptide motifs that are proline-rich or contain at least one proline  窗体底端 | 17 | ICC | 2/55 |

**Supplemental Table 4: IC50 values of different fusion chimeras to target drugs**

| **Cell lines** | **Target drugs** | **Chimeras** | **IC50(nM)** |
| --- | --- | --- | --- |
| RBE | AZD4547 | GFP-Control | ˃ 1000 |
|  |  | BF846 | ˃ 1000 |
|  |  | FB949 | 113.3 |
|  |  | BF494 | 130.2 |
|  |  | BF494-Truncated | 145.8 |
|  |  | BF494- Mutation | ˃ 1000 |
|  | BGJ398 | GFP-Control | ˃ 1000 |
|  |  | BF846 | ˃ 1000 |
|  |  | FB949 | 70.8 |
|  |  | BF494 | 69.5 |
|  |  | BF494-Truncated | 123.7 |
|  |  | BF494- Mutation | ˃ 1000 |
| HCCC-9810 | AZD4547 | GFP-Control | ˃ 1000 |
|  |  | BF846 | ˃ 1000 |
|  |  | FB949 | 177.6 |
|  |  | BF494 | 418.4 |
|  |  | BF494-Truncated | 52.2 |
|  |  | BF494- Mutation | ˃ 1000 |
|  | BGJ398 | GFP-Control | ˃ 1000 |
|  |  | BF846 | ˃ 1000 |
|  |  | FB949 | 180.7 |
|  |  | BF494 | 92.8 |
|  |  | BF494-Truncated | 87.5 |
|  |  | BF494- Mutation | ˃ 1000 |

**Supplemental Figure 1. Expression of fusion chimeras.**

The cDNAs of *FGFR2* fusion chimeras were transfected into RBE cells. The expression of chimeras was detected by Western blot with anti-Flag or anti-*FGFR2* antibody.

**Supplemental Figure 2. Colony formation activity of all FGFR2 fusions.**

Representative image of colonies expressing all FGFR2 fusions in NIH3T3 cells are shown (scale bar=100 μm) (a). Average colonies with all of FGFR2 fusions are plotted. *P<0.05 (b).

**Supplemental Figure 3. Transwell migration activity of all FGFR2 fusions.**

Representative image of transwell migration expressing all FGFR2 fusions in RBE cells (a) and HCCC-9810 are shown (c) (scale bar=100 μm). Average number of migrated cells with all FGFR2 fusions in RBE cells (b) and HCCC-9810(d) are plotted.

**Supplemental Figure 4. Proliferation activity of all FGFR2 fusions in Ba/F3 cells with IL3.** Growth curve of Ba/F3 cells harboring various FGFR2 fusions in the presence of IL3 are shown.

**Supplemental Figure 5. Proliferation activity of all FGFR2 fusions in Ba/F3 cell without IL3.** IL3-independent growth curve of Ba/F3 cells harboring various FGFR2 fusions are shown.

**Supplemental Figure 6. Response of HCCC-9810 cells expressing *FGFR2* fusions to *FGFR2*-selective SMKIs BGJ398 and AZD4547.**

Dose-response curves for BGJ398 (a) and AZD4547 (b) in HCCC-9810 cells. The viability of HCCC-9810 cells expressing different FGFR2 fusions was detected by CCK8 assay after treatment with indicated concentration of BGJ398 or AZD4547.

**Supplemental Figure 7. Response of HCCC-9810 cells expressing BF494** [**variation**](http://www.baidu.com/link?url=rni4j1US4-qX99fpIkTv30IS2pwWfjqudcgQG1C6zLF4AVc6jXVmYlk9wur3mR3vwupxFWpGmjUJC-Ch6CFanee6UIAVqlaZfMySRPG_NYe)**s to *FGFR2*-selective SMKIs BGJ398 and AZD4547.**

Dose-response curves for BGJ398 (a) and AZD4547 (b) in HCCC-9810 cells expressing different BF494 [variation](http://www.baidu.com/link?url=rni4j1US4-qX99fpIkTv30IS2pwWfjqudcgQG1C6zLF4AVc6jXVmYlk9wur3mR3vwupxFWpGmjUJC-Ch6CFanee6UIAVqlaZfMySRPG_NYe)s. Cell viabilities were detected by CCK8 assay.
