# Supplementary figures and images for: Typing FGFR2 translocation determines the response to targeted therapy of intrahepatic cholangiocarcinomas
Source: Cell Death Dis. 2021 Mar 11;12(3):256. doi: 10.1038/s41419-021-03548-4 (PMC7946919; doi:10.1038/s41419-021-03548-4)

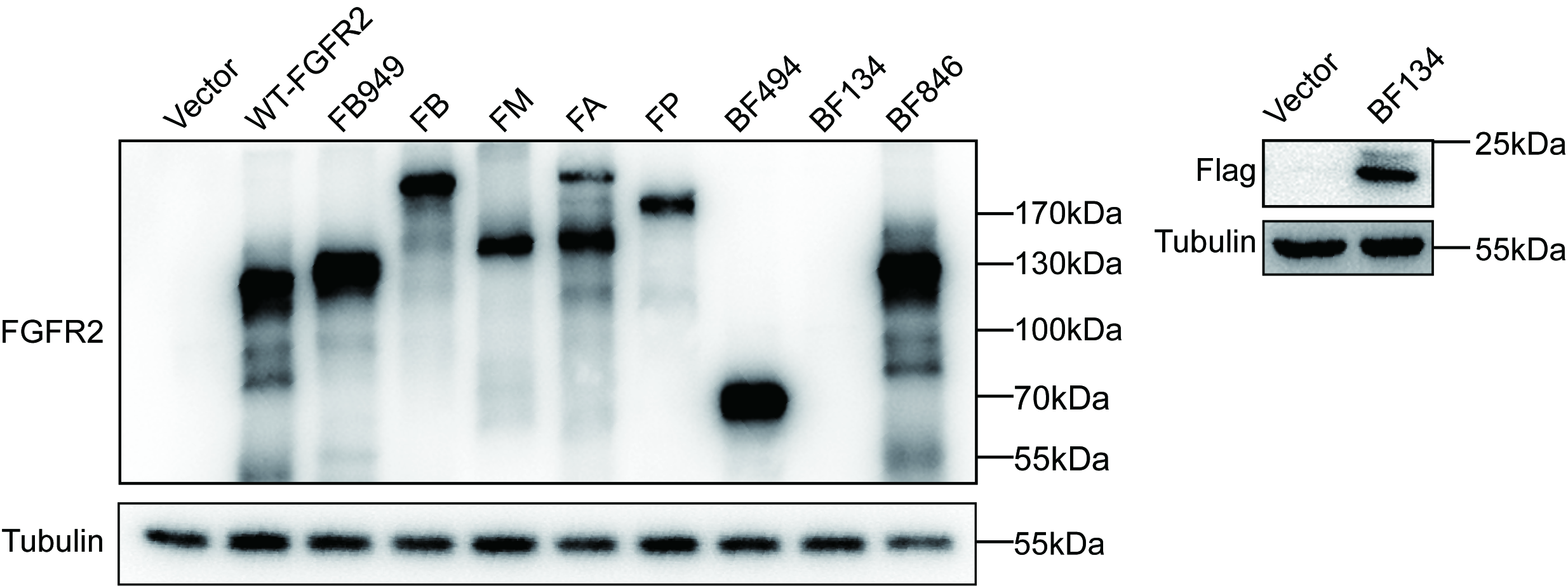

Supplement: Supplementary file 2 — supplemental figure 1 [file 41419_2021_3548_MOESM2_ESM.tif]

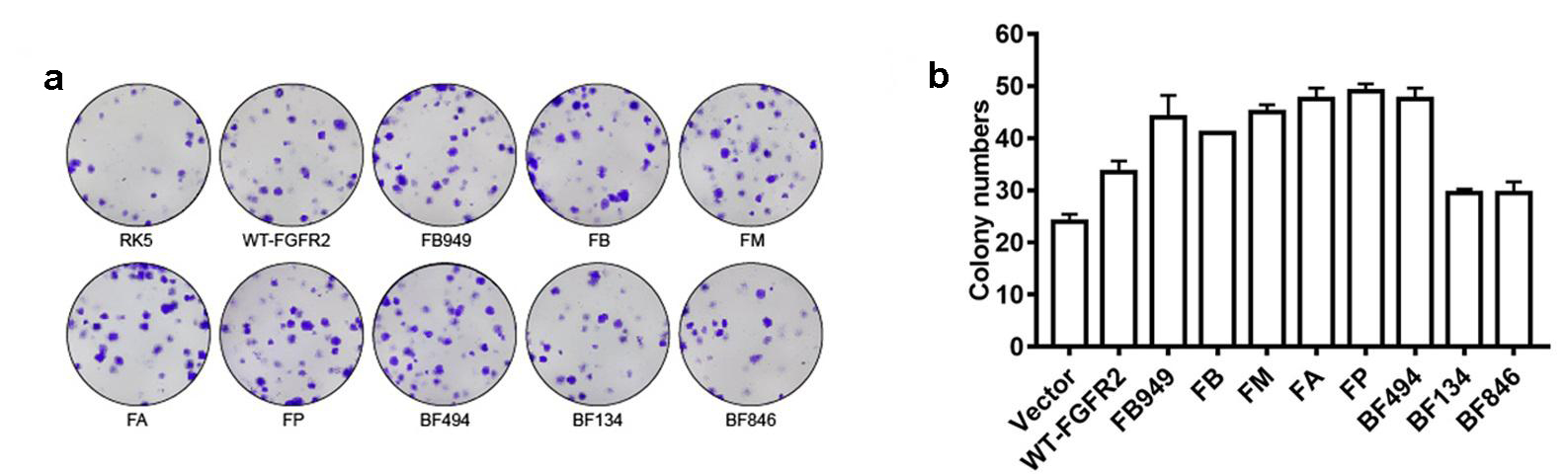

Supplement: Supplementary file 3 — supplemental figure 2 [file 41419_2021_3548_MOESM3_ESM.tif]

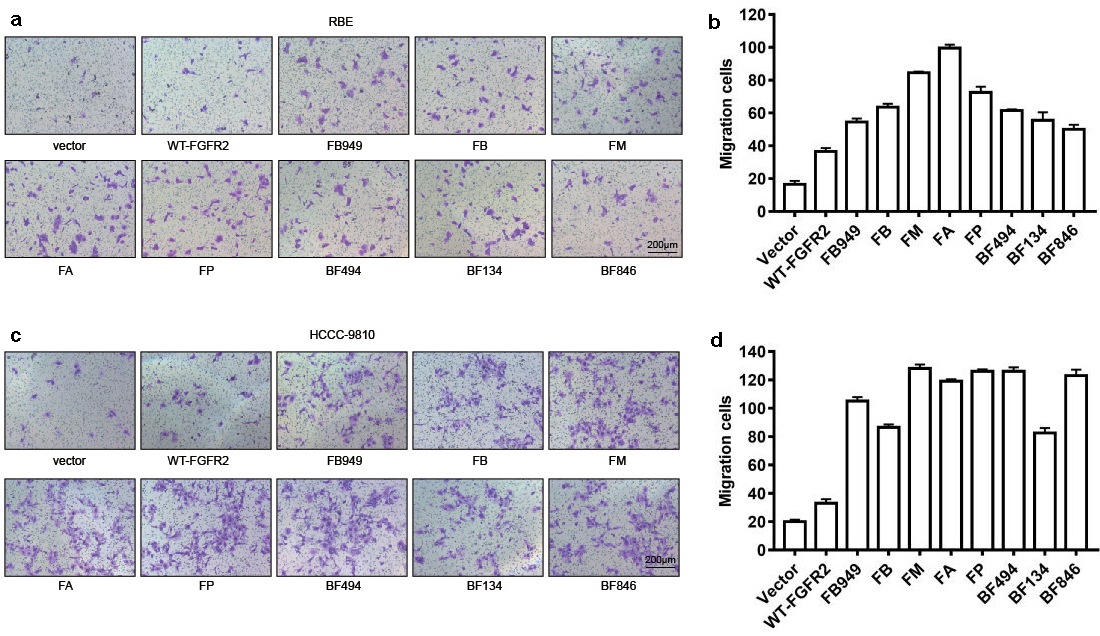

Supplement: Supplementary file 4 — supplemental figure 3 [file 41419_2021_3548_MOESM4_ESM.tif]

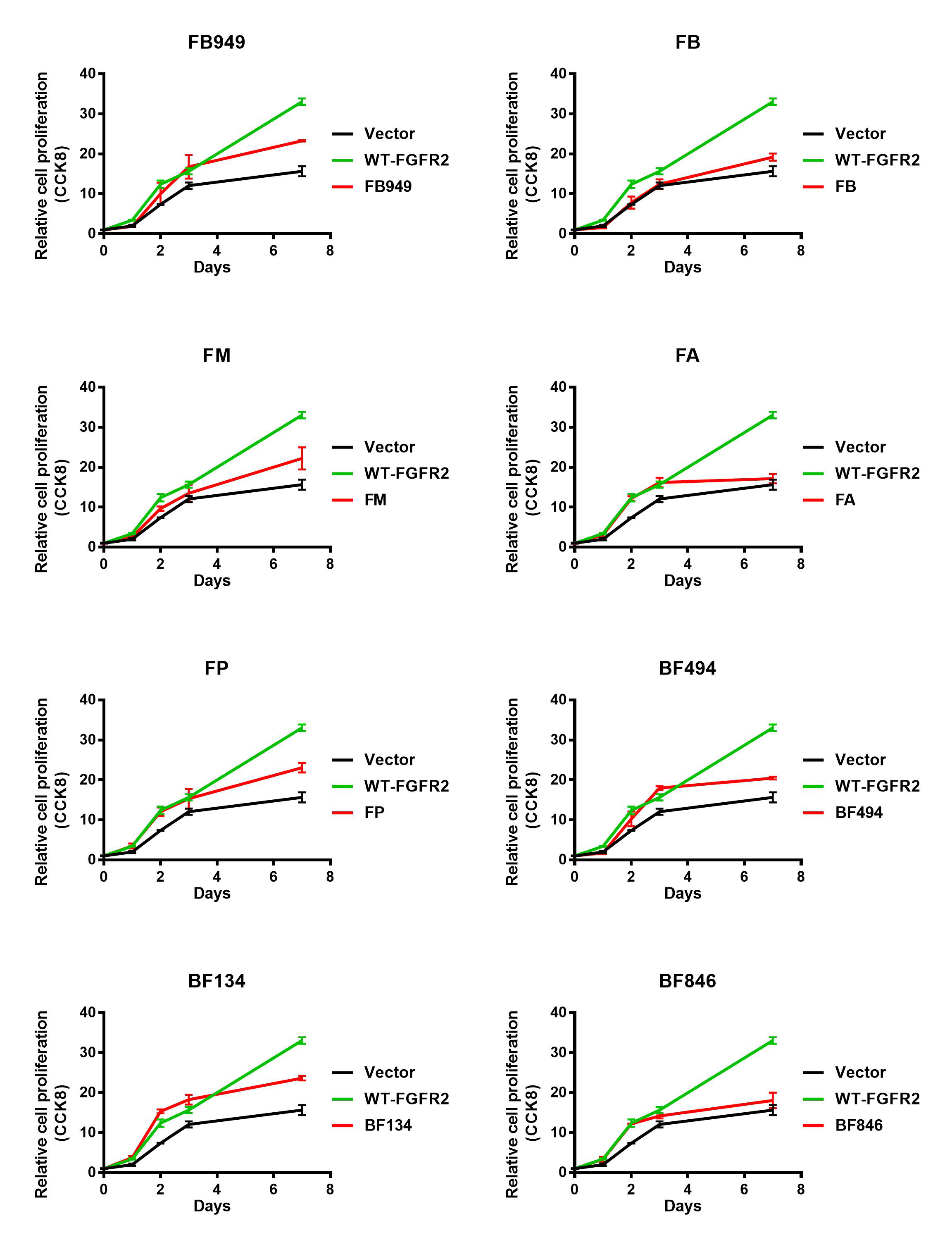

Supplement: Supplementary file 5 — supplemental figure 4 [file 41419_2021_3548_MOESM5_ESM.tif]

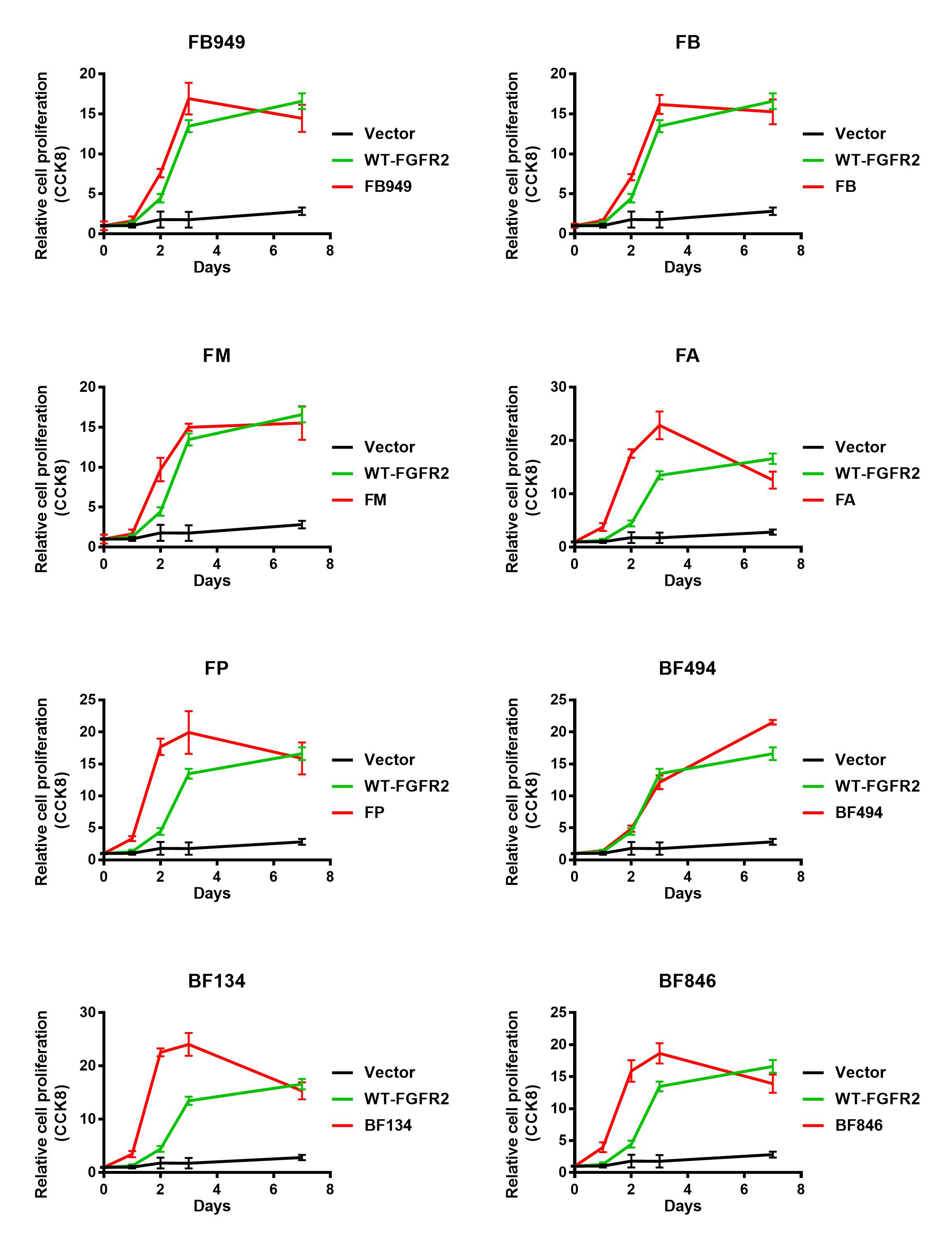

Supplement: Supplementary file 6 — supplemental figure 5 [file 41419_2021_3548_MOESM6_ESM.tif]

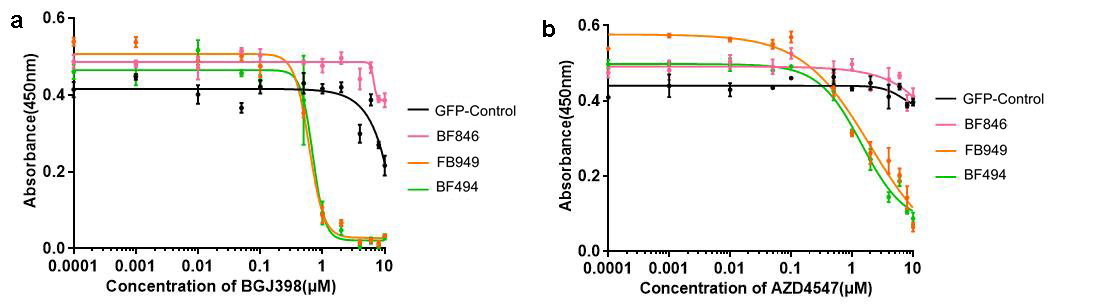

Supplement: Supplementary file 7 — supplemental figure 6 [file 41419_2021_3548_MOESM7_ESM.tif]

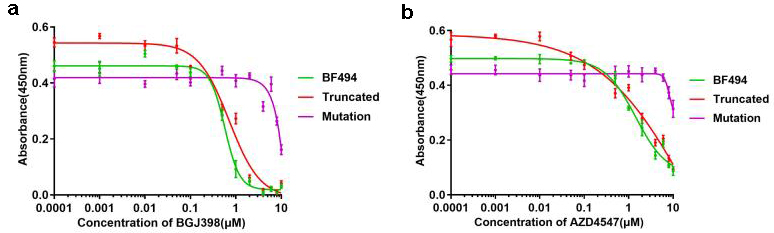

Supplement: Supplementary file 8 — supplemental figure 7 [file 41419_2021_3548_MOESM8_ESM.tif]
